# Identification of Immune Hub Genes Associated With Braak Stages in Alzheimer’s Disease and Their Correlation of Immune Infiltration

**DOI:** 10.3389/fnagi.2022.887168

**Published:** 2022-05-10

**Authors:** Xiao-hang Qian, Xiao-li Liu, Sheng-di Chen, Hui-dong Tang

**Affiliations:** ^1^Department of Neurology and Institute of Neurology, Ruijin Hospital, Shanghai Jiao Tong University School of Medicine, Shanghai, China; ^2^Department of Neurology, Shanghai Fengxian District Central Hospital, Shanghai Jiao Tong University Affiliated Sixth People’s Hospital South Campus, Shanghai, China

**Keywords:** Alzheimer’s disease, Braak stage, immune hub gene, immune cell infiltration, Tau pathology

## Abstract

**Background:**

Alzheimer’s disease (AD) is the most common type of neurodegenerative disease. Tau pathology is one of the pathological features of AD, and its progression is closely related to the progress of AD. Immune system dysfunction is an important mediator of Tau pathological progression, but the specific molecular mechanism is still unclear. The purpose of this study is to determine the immune hub genes and peripheral immune cell infiltration associated with the Braak stages, and the molecular mechanisms between them.

**Methods:**

In this study, 60 samples with different Braak stages in the GSE106241 dataset were used to screen Braak stages-related immune hub genes by using the WGCNA package in R and cytoHubba plugin. The temporal lobe expression data in the Alzdata database were used to verify the results. The correlation between the expression level of immune core genes and the pathological features of AD was analyzed to evaluate the abundance of peripheral immune cell infiltration and screened Braak stages-related cells. Finally, we used correlation analysis of immune hub genes and immune cells and Gene Set Enrichment Analysis (GSEA) of them.

**Results:**

Seven genes (GRB2, HSP90AA1, HSPA4, IGF1, KRAS, PIK3R1, and PTPN11) were identified as immune core genes after the screening of the test datasets and validation of independent data. Among them, Kirsten rat sarcoma viral oncogene homolog (KRAS) and Phosphoinositide-3-Kinase Regulatory Subunit 1 (PIK3R1) were the most closely related to Tau and Aβ pathology in AD. In addition, the ImmuneScore increased gradually with the increase of Braak stages. Five types of immune cells (plasma cells, T follicular helper cells, M2 macrophage, activated NK cells, and eosinophils) were correlated with Braak stages. KRAS and PIK3R1 were the immune core genes most related to the abnormal infiltration of peripheral immune cells. They participated in the regulation of the pathological process of AD through axon guidance, long-term potentiation, cytokine–cytokine receptor interaction, RNA polymerase, etc.

**Conclusion:**

The KRAS and PIK3R1 genes were identified as the immune hub genes most associated with Tau pathological progress in AD. The abnormal infiltration of peripheral immune cells mediated by these cells was involved in the Tau pathological process. This provides new insights for AD.

## Introduction

Alzheimer’s disease (AD), the most commontype of dementia, is a progressive neurodegenerative disease that is characterized by impairment in multiple cognitive domains, executive functioning disorders, and a range of neuropsychiatric symptoms (Cummings, 2021; Seto et al., 2021). Extracellular amyloid-β (Aβ) plaques and intracellular neurofibrillary tangles are the neuropathological hallmarks of AD (Busche and Hyman, 2020; Kent et al., 2020). However, the classical amyloid cascade hypothesis cannot explain all the pathological processes of AD, and there is no significant correlation between the Aβ compliance level and the cognitive level of patients with AD (Giacobini and Gold, 2013; Roda et al., 2022). More importantly, most therapeutic strategies that target different stages of the amyloid pathway have failed to achieve expected efficacy (Long and Holtzman, 2019; Mecocci and Boccardi, 2021). Another important pathological feature of AD is neurofibrillary tangles formed by the misfolding of intracellular Tau protein. This abnormal folding of Tau protein is associated with neuronal loss and synaptic dysfunction (Malpetti et al., 2020). As the disease progresses, Tau pathology spreads in a relatively stereotypic progressive pattern. According to the distribution stage, Braak et al. proposed that Tau pathology to be divided into six different stages, which are closely related to the severity of cognitive impairment and neuronal loss of patients with AD (Chung et al., 2021; Roda et al., 2022). Therefore, exploring the pathogenesis of Tau pathologic progression may provide important targets for preventing or delaying AD progression.

Recently, the important role of immune system dysfunction in aging or neurodegenerative diseases has attracted extensive attention (Passaro et al., 2021). In the central nervous system (CNS), microglia are the most important innate immune cells. They originate from myeloid progenitors in the yolk sac and play a physiological role in the clearance of abnormal aggregates, signal transduction, maintenance of homeostasis, and synaptic plasticity (Cisbani and Rivest, 2021). In AD, microglia can be activated by misfolded proteins and participate in a series of pathological processes, such as neuroinflammatory initiation, Aβ aggregation, and neuron loss (Ennerfelt and Lukens, 2020; Qian et al., 2021). Recent studies on patients with AD and animal models have proved that microglia are important vectors in the transmission of Tau pathology (Hopp et al., 2018; Pascoal et al., 2021). What’s more, the interaction between the peripheral immune system and the CNS also exists in AD (Passaro et al., 2021). In a healthy state, peripheral immune cells are restricted to enter the CNS by the presence of structures, such as blood-brain barrier (Greenhalgh et al., 2020). When the barrier permeability increases due to aging, trauma, infection, neurodegeneration, etc., peripheral immune cells, such as monocytes, macrophages, neutrophils, and T cells can infiltrate into the brain and affect glial and neuronal function (Greenhalgh et al., 2020). In the brain of patients with AD, extravascular T cells were detected, specifically in the hippocampus, and the abundance of T cells was correlated with tau pathology without Aβ pathology (Merlini et al., 2018). Another study confirmed that T cells infiltration abundance in the brain of patients with AD was positively correlated with *p*-Tau levels (Zotova et al., 2013). These data strongly suggest a close relationship between immune system dysfunction and AD-associated Tau pathological process. However, understanding the molecular biological mechanism of immune system-driven abnormal Tau propagation accelerates AD progression remains unclear.

In this study, we used weighted gene co-expression network analysis to identify immune hub genes closely associated with Braak stages in AD and then validated by using independent datasets. In addition, we analyzed the abundance of peripheral immune cell infiltration in the brain associated with Braak stages in AD through the CIBERSORT algorithm. Finally, the correlation between immune hub genes and the abundance of peripheral immune cell infiltration was analyzed. This study will provide an important basis for exploring the cellular and molecular mechanisms related to the Tau pathological process of AD from the perspective of immunology.

## Materials and Methods

### Data Collection and Processing

The GSE106241 data file was downloaded from the NCBI Gene Expression Omnibus public database (GEO, https://www.ncbi.nlm.nih.gov/geo/) annotated by GPL24170 as a Series Matrix File. The dataset included data on gene expression profiles from 60 human temporal cortical tissue samples with varying degrees of AD-related neurofibrillary pathology. The AD pathological features, such as Braak stages, α, β, and γ—secretase activity, and Aβ_1–42_ levels of each sample were downloaded from the GEO database to perform Pearson’s correlation analysis with Braak stages-related immune hub genes (https://ncbi.nlm.nih.gov/geo/geo2r/?acc=GSE106241). The AlzData database was a full collection of current high-throughput omics databases, such as genomics (GWAS and Whole Exome Sequencing), Proteomics, Functional genomics, and Transcriptomes data (Xu et al., 2018). In this study, we selected transcriptome expression data of temporal cortical from AlzData as a validation dataset, including 39 healthy controls and 52 patients with AD. In addition, the expression level of the Braak stages-related immune hub gene at the single-cell level in the brain was analyzed and visualized through the AlzData database.

### Construction of WGCNA

Based on the genetic and clinical data in GSE106241, a weighted messenger RNA (mRNA) co-expression network was constructed using the WGCNA package in R. First of all, we used the gene expression spectrum to calculate the Median Absolute Deviation (MAD) of each gene, and removed the first 50% of the smallest MAD genes. Then, we used the goodSamplesGenes method of R software package WGCNA to remove outlier genes and samples. WGCNA was further used to build a scale-free co-expression network. After the acquisition of an appropriate power (β = 6), the adjacency matrix was transformed into the topological overlap matrix (TOM). Third, hierarchical clustering was performed to identify modules, and the eigengene was calculated. Finally, we calculated the correlation between Braak stages and each module through Pearson’s correlation analysis.

### Gene Ontology Functional, Kyoto Encyclopedia of Genes and Genomes Pathway Enrichment, and Protein-Protein Interaction Network Analysis

Functional enrichment was analyzed through the STRING online tool to investigate GO cellular components (CC), biological process (BP), molecular function (MF), and Kyoto Encyclopedia of Genes and Genomes (KEGG) pathways related to potential immune-related pathogenesis of Braak stages in AD. The interaction score was 0.4. The false discovery rate (FDR) was < 0.05. The protein-protein interaction (PPI) network was constructed through the NetworkAnalyst based on the STRING interactome with a 900 confidence score (https://www.networkanalyst.ca/) (Xia et al., 2015).

### Immune-Related Hub Genes Selection and Validation

Then, the total of 2,483 immune-related genes list was download from the Immunology Database and Analysis Portal (ImmPort) (https://www.immport.org/home) to screen out the DEIRGs (Bhattacharya et al., 2014). The cytoHubba plugin was adopted to screen out immune-related hub genes through three different algorithms [Edge Percolated Component (EPC), Maximum Neighborhood Component (MNC), and Degree]. In total, 91 temporal lobe transcriptomic data (39 healthy controls and 52 patients with AD) from the AlzData database were used as a validation dataset to analyze the difference in immune hub genes between the AD and HC groups.

### Immune Cell Infiltration Abundance Analysis

In this study, CIBERSORT was used to assess the abundance of 22 types of immune cells in 60 samples with different Braak stages. CIBERSORT is an analytical algorithm that uses normalized gene expression profiles to assess the abundance of specific cells in complex tissues (Newman et al., 2015). After evaluating the abundance of 22 types of immune cells in each sample, we performed differential analysis and correlation analysis according to the Braak stages of the samples.

### Gene Set Enrichment Analysis

The Gene Set Enrichment Analysis (GSEA) was used to identify the different signal pathways between the high and low levels of immune hub genes in GSE106251. The annotated gene set c2.cp.kegg.v7.1.symbols.gmt was chosen as the reference gene list. The cut-off value for the GSEA was set as *p* < 0.05.

### Statistical Analysis

Statistical analysis and graphs were performed using Sangerbox online software (http://sangerbox.com/) and GraphPad Prism 5.0 software. A value of *p* less than 0.05 was considered statistically significant. Multiple testing corrections were made using the Bonferroni correction and Duncan’s multiple range test.

## Results

### WGCNA Was Established to Screen Genes Associated With Braak Stages

**FIGURE 1:**
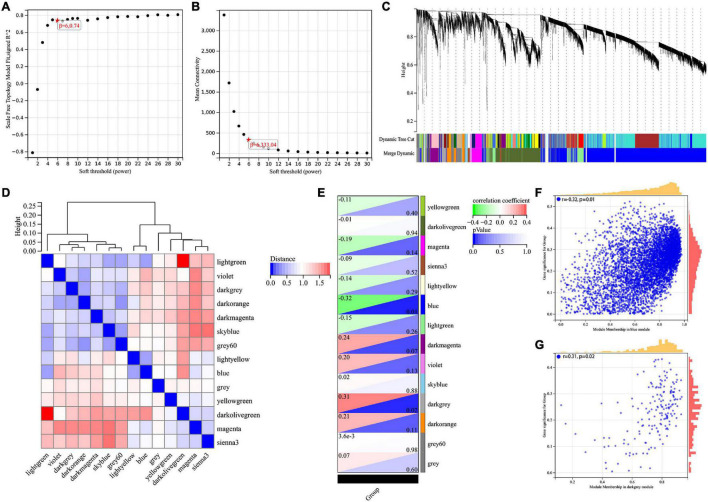
Screening for BRAAK staging related genes by WGCNA. **(A)** Analysis of the scale-free index for various soft-threshold powers (β). **(B)** Analysis of the mean connectivity for various soft-threshold powers. **(C)** Recognition module, each module was given an individual color as identifier, such as 14 different modules. **(D)** Co-expression similarity of entire modules based on the hierarchical clustering of module eigengenes and the correlation between different modules, red indicates high adjacency (positive correlation) and blue low adjacency (negative correlation). **(E)** In the correlation heat map of gene modules and phenotypes, red is positively correlated with the phenotype; green is negatively correlated with the phenotype. **(F,G)** Scatter plots for correlations between gene significance and module membership in blue and darkgrey module.

The WGCNA method was used to identify genes associated with Braak stages. First of all, we screened the top 50% highest variance of the expression profile (a total of 9,386 genes) from 60 samples for WGCNA analysis. Then, the scale-free network was constructed with a β value equal to 6 (*R*^2^ = 0.74) (Figures 1A,B). Finally, a total of 14 co-expression modules were identified (Figure 1C). The connectivity was calculated and cluster analysis was performed among the 14 modules (Figure 1D). To further analyze the association between the models and phenotype, we calculated the correlation coefficients of each model with Braak stages. The results showed that the blue module (*r* = −0.32, *p* = 0.01) was the most negatively and the dark gray module (*r* = 0.31, *p* = 0.02) was the most positively associated with the Braak stage (Figures 1E–G). In total, 5,374 genes from these two modules were selected for the next analysis.

### Enrichment Analysis of Braak Stages-Related Immune Gene in AD and PPI Network Analysis

**FIGURE 2:**
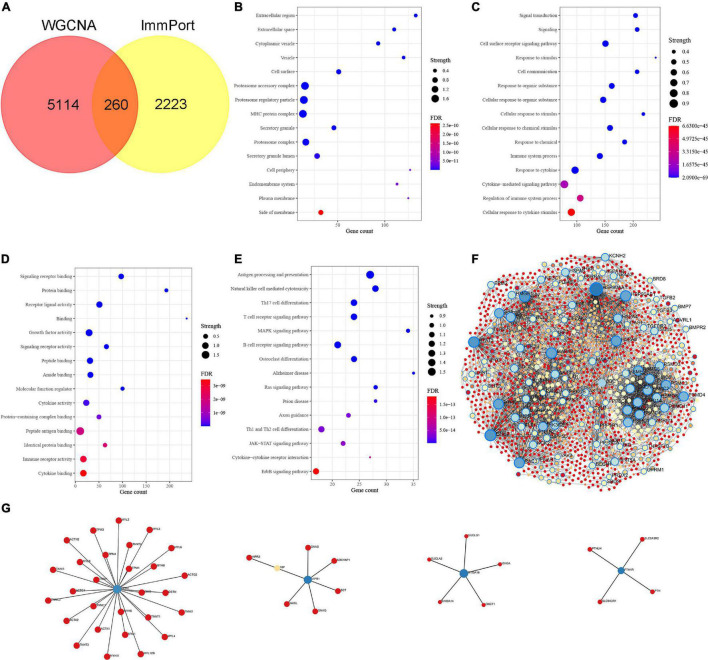
Identification of Braak stages associated immune genes and enrichment analysis of Gene Ontology (GO) and Kyoto Encyclopedia of Genes and Genomes (KEGG). **(A)** The Venn diagram shows the common Braak stages associated immune genes between the Braak stages associated genes and the ImmPort dataset. **(B–D)** GO biological function enrichment analysis. **(E)** KEGG pathway enrichment analysis. **(F,G)** Protein-protein interaction (PPI) network of Braak stages associated immune genes. FDR, false discovery rate.

We intersected the above Braak stages-related immune genes screened by WGCNA with immune gene in the ImmPort database to screen out Braak stages-related immune genes. Among them, there were 260 Braak stages-related immune genes (Figure 2A and Supplementary Table 1). The enrichment analysis of GO cellular components revealed that these genes were mainly located at the extracellular region, cell surface, proteasome complex, MHC protein complex, etc. (Figure 2B). The biological processes of each of them were associated with signal transduction, cytokine-mediated signaling pathway, response to cytokine, cellular response to cytokine stimulus, etc. (Figure 2C). The enrichment analysis of GO molecular function showed that Braak stage-related immune genes were involved in signaling receptor binding, growth factor activity, cytokine activity, peptide antigen binding, etc. (Figure 2D). The KEGG enrichment analysis revealed that these genes were involved in AD, antigen processing and presentation, natural killer cell-mediated cytotoxicity, B-cell receptor signaling pathway, etc. (Figure 2E). The PPI network of the 260 Braak stages-related immune genes was constructed (Figures 2F,G). These results strongly suggest the role of immune-related genes in the pathological progression of Braak stages in AD.

### Screening of Braak Stages-Related and Validation in Database

**FIGURE 3:**
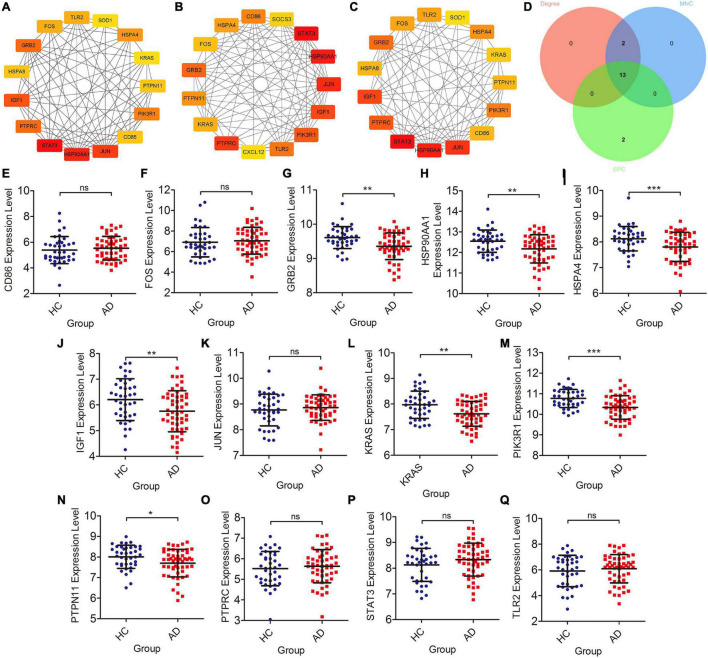
Screening of immune hub genes and database validation of their expression levels. **(A–C)** The top 15 immune hub genes identified by MNC, EPC, and Degree algorithms in cytoHubba plugin. **(D)** Venn diagram showing the intersection of immune hub genes obtained by the three algorithms. **(E–Q)** The different expression levels of immune hub genes in the temporal cortex between the AD and HC groups were validated in the Alzdata database. *p < 0.05, **p < 0.01, ***p < 0.001, and ns, no sense.

The cytoHubba plugin was adopted to screen out Braak stages-related immune hub genes through three different algorithms (EPC, MNC, and Degree). The top 15 hub genes, filtered by the EPC algorithm, were CD86, HSPA4, FOS, GRB2, PTPN11, Kirsten rat sarcoma viral oncogene homolog (KRAS), PTPRC, CXCL12, TLR2, Phosphoinositide-3-Kinase Regulatory Subunit 1 (PIK3R1), IGF1, JUN, HSP90AA1, STAT3, and SOCS3 (Figure 3A). The DEGREE screened out TLR2, FOS, GRB2, HSPA8, IGF1, PTPRC, STAT3, HSP90AA1, JUN, CD86, PIK3R1, PTPN11, KRAS, HSPA4, and SOD1 (Figure 3B). TLR2, FOS, GRB2, HSPA8, IGF1, PTPRC, STAT3, HSP90AA1, JUN, CD86, PIK3R1, PTPN11, KRAS, HSPA4, and SOD1were found out by the MNC (Figure 3C). Finally, the co-existing gene of the three algorithms was selected as the hub gene, such as CD86, HSPA4, FOS, GRB2, KRAS, PTPN11, PTPRC, TLR2, PIK3R1, IGF1, JUN, HSP90AA1, and STAT3 (Figure 3D). After that, we then validated these Braak stages-related immune hub genes by using the temporal lobe transcriptome data of 39 healthy controls and 52 patients with AD from the Alzdata database. The results showed that the expression levels of GRB2, HSP90AA1, HSPA4, IGF1, KARS, PIK3R1, and PTPN11 were significantly decreased in the AD group compared with the HC group (Figures 3G–J,L–N), and the expression levels of CD86, FOS, JUN, PTPRC, STAT3, and TLR2 were not statistically different between the AD and HC groups (Figures 3E,F,K,O–Q).

### Correlation Analysis of Braak Stages-Related Immune Hub Genes With Pathological Features of AD

**FIGURE 4:**
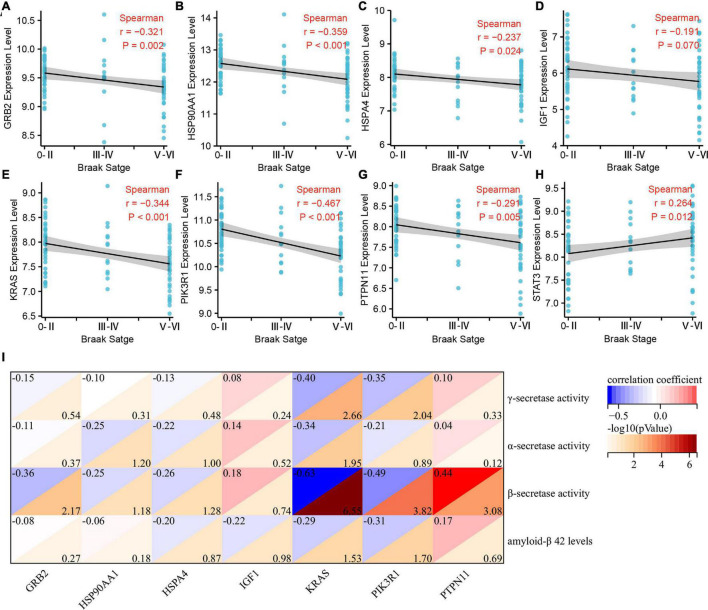
Correlation analysis of Braak stages-related immune hub genes with pathological features of Alzheimer’s disease (AD). **(A–H)** Correlation analysis between the expression level of immune hub genes and Braak stages. **(I)** Correlation analysis between the expression level of immune hub genes and α, β, γ-secretase activity, and Aβ_1–42_.

We further analyzed the correlation between Braak stages-related immune core genes and AD pathological features, such as Braak stages, α, β, γ-secretase activity, and Aβ_1–42_ levels. The expression levels of GRB2 (*r* = −0.321, *p* = 0.002), HSP90AA1 (*r* = −0.359, *p* < 0.001), IGF1 (*r* = −0.191, *p* = 0.07), KRAS (*r* = −0.344, *p* < 0.001), PIK3R1 (*r* = −0.467, *p* < 0.001), and PTPN11 (*r* = −0.291, *p* = 0.005) were negatively correlated with the grade of Braak stages (Figures 4A–G). The expression level of STAT3 was positively correlated with Braak stages (*r* = 0.264, *p* = 0.012) (Figure 4H). In amyloidogenic APP processing, we found that the γ-secretase activity was negatively correlated with the expression level of KARS (*r* = −0.40, *p* = 0.002) and PIK3R1 (*r* = −0.35, *p* = 0.009). The β-secretase activity was negatively correlated with the expression level of GRB2 (*r* = −0.36, *p* = 0.006), KRAS (*r* = −0.63, *p* < 0.001), PIK3R1 (*r* = −0.49, *p* < 0.001), and was positively correlated with PTPN11 expression level (*r* = 0.44, *p* < 0.001). In addition, the Aβ_1–42_ levels were negatively correlated with KRAS (*r* = −0.29, *p* = 0.029) and PIK3R1 (*r* = −0.31, *p* = 0.019). However, the expression level of KRAS (*r* = −0.34, *p* = 0.011) was negatively correlated with the α-secretase activity in non-amyloidogenic APP processing (Figure 4I). Accordingly, we found that KRAS and PIK3R1 were not only involved in the Tau pathologically related Braak stages, but also closely related to the regulation of Aβ pathology.

### Abundance of Immune Cell Infiltration in Patients With AD With Different Braak Stages

**FIGURE 5:**
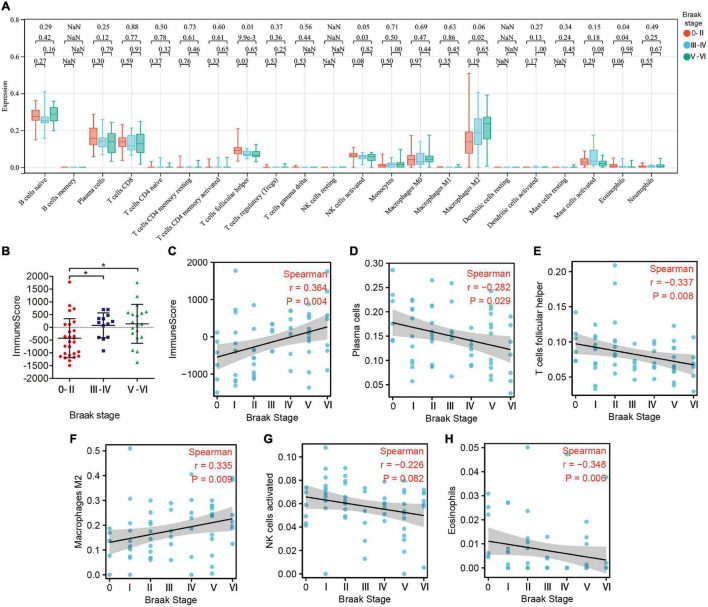
The landscape of immune cell infiltration in different Braak stages. **(A)** The box plot shows the difference of 24 immune cell proportions between different Braak stages. Red color represents Braak 0-II, blue color represents Braak III-IV, and green color represents Braak V-VI. The values of *p* between each group were shown at the top of the panel **(A)**. **(B)** Differences in ImmuneScores between Braak stages. **(C–H)** Correlation analysis between differential immune cell abundance and Braak stages.

Subsequently, we estimated the abundance of 22 kinds of immune cell infiltration in the GSE106241 samples by CIBERSORT to explore the role of the peripheral immune system in the progression of Braak stages in AD. We found that the ImmuneScore, which represents the total level of immune cells infiltration, increased with the increase of Braak stages (Figures 5B,C). In immune cell subtype analysis, four kinds of immune cells were significantly different in different Braak stages (Figure 5A). Among them, the Braak stages were negatively correlated with the abundance of follicular helper T cells (*r* = −0.337, *p* = 0.008), activated NK cells (*r* = −0.226, *p* = 0.082), and eosinophils (*r* = −0.348, *p* = 0.008) (Figures 5E,G,H). The abundance of M2 macrophages was positively correlated with Braak stages (Figure 5F). In addition, the abundance of plasma cells was negatively correlated with Braak stages (Figure 5D). Therefore, we speculated that peripheral immune cells play an important role in the pathological process of AD.

### Correlation Analysis of Braak Stages-Related Immune Hub Genes and Immune Cells

**FIGURE 6:**
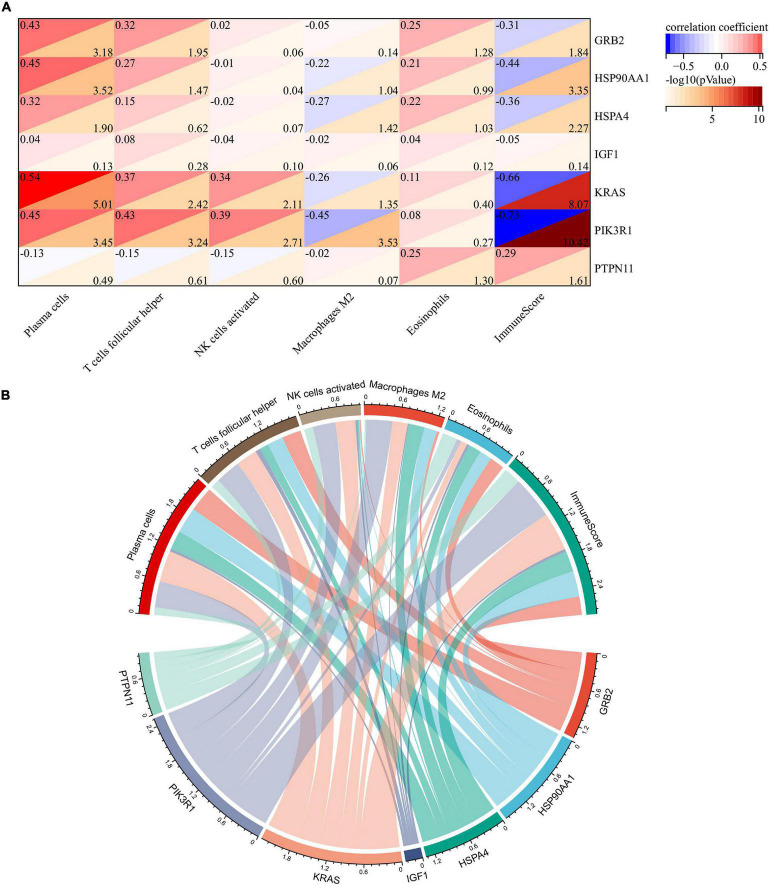
Correlation analysis of immune hub genes with different infiltrating immune cells. **(A)** Heatmap of correlation between immune hub genes and different infiltrating immune cells. **(B)** Network diagram of interactions between immune hub genes and different infiltrating immune cells. The larger the circle represents the greater interaction with other.

To explore whether these two core genes were involved in regulating abnormal infiltration of peripheral immune cells, we conducted a correlation analysis between immune hub genes and differentially infiltrated immune cells (Figures 6A,B). The ImmuneScore was negatively correlated with GRB2 (*r* = −0.31, *p* = 0.014), HSP90AA1 (*r* = −0.44, *p* < 0.001), HSPA4 (*r* = −0.36, *p* = 0.005), KRAS (*r* = −0.66, *p* < 0.001), and PIK3R1 (*r* = −0.73, *p* < 0.001) expression level, and was positively correlated with the PTPN11 (*r* = 0.29, *p* = 0.025). The abundance of plasma cells was positively correlated with GRB2 (*r* = 0.43, *p* < 0.001), HSP90AA1 (*r* = 0.45, *p* < 0.001), HSPA4 (*r* = 0.32, *p* = 0.013), KRAS (*r* = 0.54, *p* < 0.001), and PIK3R1 (*r* = 45, *p* < 0.001) expression levels. The abundance of follicular helper T cells was positively correlated with GRB2 (*r* = 0.32, *p* = 0.011), HSP90AA1 (*r* = 0.27, *p* = 0.034), KRAS (*r* = 0.37, *p* = 0.004), and PIK3R1 (*r* = 43, *p* < 0.001) expression levels. The abundance of activated NK cells was positively correlated with KRAS (*r* = 0.34, *p* = 0.008) and PIK3R1 (*r* = 0.43, *p* = 0.002). The abundance of M2 macrophage was negatively correlated with HSPA4 (*r* = −0.27, *p* = 0.038), KRAS (*r* = −0.26, *p* = 0.045), and PIK3R1 (*r* = −0.45, *p* < 0.001). Among them, we found that KRAS and PI3KR1 were the two most important hub genes in Braak stages-related immune regulation (Figure 6B).

### Single Cell Expression Level Detection and GSEA for Braak Stages Related Immune Hub Gene

**FIGURE 7:**
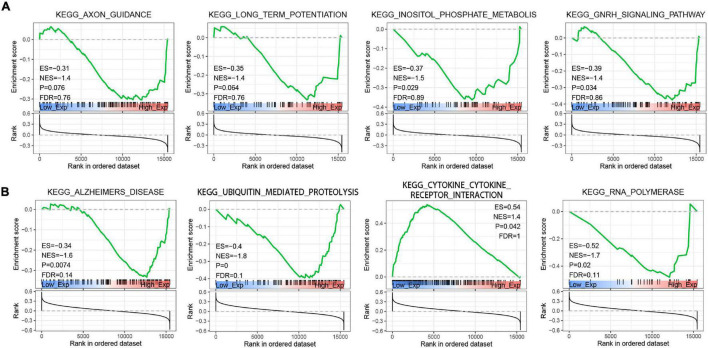
Gene Set Enrichment Analysis (GSEA) screened the potential molecular mechanism of immune core genes. **(A)** GSEA of Kirsten rat sarcoma viral oncogene homolog (KRAS). **(B)** GSEA of Phosphoinositide-3-Kinase Regulatory Subunit 1 (PIK3R1). ES, Enrichment Score; NES, Normalized Enrichment Score; and FDR, false discovery rate.

To explore the function of Braak stages-related immune hub genes, we analyzed the expression level at the single-cell level of brain tissue. The results showed that the expression level of KRAS was the highest in neurons, but was limited in the other five cell types (endothelial, astrocytes, microglia, oligodendrocytes, and oligodendrocyte precursor cell) (Supplementary Figure 1A). The expression level of PIK3R1 was relatively high in six kinds of cells, among which the expression level was highest in neurons and astrocytes (Supplementary Figure 1B). After that, we explored the potential molecular mechanisms of KRAS and PI3KR1 associated with Braak stages in AD through GSEA. The results showed that axon guidance, long-term potentiation, inositol phosphate metabolism, and GnRH signaling pathway were significantly enriched in groups with high KRAS expression (Figure 7A). In addition, the high expression of PI3KR1 is involved in AD and Ubiquitin mediated proteolysis (Figure 7B). The PI3KR1 low expression group was related to cytokine–cytokine receptor interaction and RNA polymerase (Figure 7B). These results suggested that both KRAS and PI3KR1 were involved in the pathway of AD-related pathological mechanisms.

## Discussion

Alzheimer’s disease, the most common form of dementia, currently has limited therapeutic options. Aβ and Tau are two typical pathological features of AD. Most previous studies on therapeutic strategies for AD have focused on the amyloid pathway. But almost all these studies ended in failure. In addition, the Tau pathological degree is closely related to the cognitive impairment level of patients with AD, while Aβ pathology cannot reflect the severity of patients with AD (Chung et al., 2021; Roda et al., 2022). In recent years, the role of the immune system in regulating the progress of AD was the latest hotspot. Pascoal et al. (2021) first confirmed the role of activated microglia in the spatial transmission of Tau protein in the brain of patients with AD by positron emission tomography (PET). However, it is not only the central innate immune cells but also the peripheral immune system that are critical for the maintenance of the CNS homeostasis and the progress of AD (Jevtic et al., 2017; Castellani and Schwartz, 2020). Therefore, exploring the molecular mechanism of immune system progression in Tau pathology in patients with AD will provide new strategies for treatment.

In the present study, we identified thirteen immune hub genes (CD86, HSPA4, FOS, GRB2, KRAS, PTPN11, PTPRC, TLR2, PIK3R1, IGF1, JUN, HSP90AA1, and STAT3) associated with Braak stages in AD through WGCNA and the cytoHubba plugin. After verifying the expression level in independent datasets and correlation analysis of AD pathological features, KRAS and PI3KR1b were finally identified as the most reliable Braak stages-associated immune hub genes. KRAS was the most common oncogene. The mutations of the KRAS gene can accelerate and maintain tumorigenesis (Mustachio et al., 2021). However, its role in neurodegenerative diseases has been limited. In our results, the expression level of KRAS was significantly decreased in patients with AD, and was negatively correlated with Braak stages and Aβ pathology. In APP/PS1 mice, the expression level of Kras was also decreased (Xiao et al., 2021). The GSEA showed that the axon guidance and long-term potentiation were enriched in the high expression level of KRAS. This was consistent with the result that KRAS was highly expressed in neurons. A previous study reported that KRAS was selected as putative neuronal cell cycle re-entry related factor in AD (Yuen et al., 2022). In BV-2 cells, Aβ can induce cell apoptosis by decreasing KRAS expression levels (Xiao et al., 2021). These results suggested the function of KRAS in regulating the cell cycle and promoting cell proliferation in the brain. However, there was no significant correlation between the immune signaling pathway and KRAS in GSEA analysis, which may be related to the dataset selected in this study. PIK3R1 was a member of the class IA in the PI3K family, which took part in the regulation of cell proliferation, differentiation, survival, etc. (Huang et al., 2020). Genome-wide network analysis has reported that the PIK3R1 was associated with Aβ production in AD (Cong et al., 2017). Moreover, the PIK3R1 polymorphism (Met326Ile) was closely associated with the genetic susceptibility of female patients with AD patients, which may be related to interference with insulin signals in the brains of patients with AD (Liolitsa et al., 2002). In this study, we identified PIK3R1 as a BRAAK stage-associated immune hub gene. Its expression level was decreased in patients with AD and negatively correlated with BRAAK stage and Aβ pathology. In the brain, PIK3R1 was widely expressed, such as endothelial, astrocyte, microglia, oligodendrocyte, OPC, and neuron. The GSEA showed that AD, ubiquitin-mediated proteolysis, and RNA polymerase were enriched in the high PIK3R1 expression level AD group. In addition, our result showed that PIK3R1was involved in the regulation of immunity through cytokine–cytokine receptor interaction. More importantly, previous studies have reported that heterozygous mutation in PIK3R1 lead to activated phosphoinositide 3-kinase delta syndrome (APDS), which is a primary immunodeficiency and immune dysregulation (Nunes-Santos et al., 2019). It is speculated that the low expression level of PIK3R1 in AD may affect the function of immune cells, such as microglia. However, the specific mechanisms of KRAS and PIK3R1 in AD need to be further verified *in vivo* or *in vitro*.

However, due to the limitations of previous research techniques, it is difficult to conduct a relative quantitative analysis of peripheral immune cells in the CNS of AD and further study their mechanism of action. In this study, the CIBERSORT was used to evaluate the relative abundance of immune cell infiltration associated with Braak stages in patients with AD. Our results showed that the ImmuneScore increased with Braak stages, which suggested the chronic activation of the immune system in patients with AD. Among the 22 immune cell subtypes, we found that five types of immune cell abundance were associated with Braak stages, such as plasma cells, T follicular helper cells, M2 macrophage, activated NK cells, and eosinophils. T follicular helper cells and plasma cells were adaptive immune cells. Previous studies have shown that T follicular helper cells can assist B cells to perform effector humoral immunity (Gowthaman et al., 2021). This may explain the reduced consistency of T follicular helper cells and plasma cells with high Braak stages group in our results. In addition, the abundance of activated NK cells was also decreased in the high Braak stages group. In the 5XFAD model, the absence of B cells, T cells, and NK cells can accelerate the disease progression (Marsh et al., 2016). As for eosinophils, our results confirmed that eosinophil abundance was inversely associated with the Braak stage. Järemo et al. (2013) reported a reduction in the number of eosinophils in the peripheral blood of patients with AD, which was consistent with our results in the brain. A recent study reported that the eosinophils have a protective effect on maintaining normal physiological function and immune homeostasis in old age reported recently (Brigger et al., 2020). However, our study only analyzed the correlation between these different peripheral immune cells and the pathological characteristics of AD, and the specific role and potential mechanism of each immune cell in AD need further study. Furthermore, we found that KRAS and PIK3R1 were the genes most closely associated with peripheral immune cell infiltration. KRAS and PIK3R1 were negatively correlated with ImmuneScore and M2- macrophage abundance but positively correlated with plasma cells, T follicular helper cells, activated NK cells, and eosinophils. However, in this study, we did not detect the expression levels of KRAS and PIK3R1 in peripheral immune cells in the brain. This may be related to the low abundance of peripheral infiltrating immune cells in the brain. Further studies are needed to explore the molecular mechanisms by which they regulate immune cells in AD.

In summary, we identified KRAS and PIK3R1 as Braak stages-associated immune hub genes in AD. They were also correlated with Aβ pathology. In addition, this study indicated that the abundance of plasma cells, T follicular helper cells, M2 macrophage, activated NK cells, and eosinophils were related to the progression of Braak stages in AD. Besides, KRAS and PIK3R1 were negatively correlated with ImmuneScore and M2- macrophage abundance but positively correlated with plasma cells, T follicular helper cells, activated NK cells, and eosinophils. Further exploration of Braak stages-related immune genes and the role of differential infiltrating immune cells in the progression of AD will provide new targets for the pathogenesis and treatment of AD.

## Data Availability Statement

Publicly available datasets were analyzed in this study. This data can be found here: https://ncbi.nlm.nih.gov/geo/query/acc.cgi?acc=GSE106241.

## Author Contributions

H-DT and S-DC designed the study and prepared the manuscript. X-HQ and X-LL developed the methodology and analyzed the data. All authors discussed the results and approved the manuscript.

## Conflict of Interest

The authors declare that the research was conducted in the absence of any commercial or financial relationships that could be construed as a potential conflict of interest.

## Publisher’s Note

All claims expressed in this article are solely those of the authors and do not necessarily represent those of their affiliated organizations, or those of the publisher, the editors and the reviewers. Any product that may be evaluated in this article, or claim that may be made by its manufacturer, is not guaranteed or endorsed by the publisher.

## Supplementary Material

Supplementary Figure 1 Single-cell expression level of Kirsten rat sarcoma viral oncogene homolog (KRAS) and Phosphoinositide-3-Kinase Regulatory Subunit 1 (PIK3R1) in the healthy brain. **(A,B)** KRAS and PIK3R1 expression level in endothelial, astrocytes, microglia, oligodendrocytes, oligodendrocyte precursor cell (OPC), and neuron.
