# Supplementary figures and images for: Identification of Immune Hub Genes Associated With Braak Stages in Alzheimer’s Disease and Their Correlation of Immune Infiltration
Source: Front Aging Neurosci. 2022 May 10;14:887168. doi: 10.3389/fnagi.2022.887168 (PMC9129065; doi:10.3389/fnagi.2022.887168)

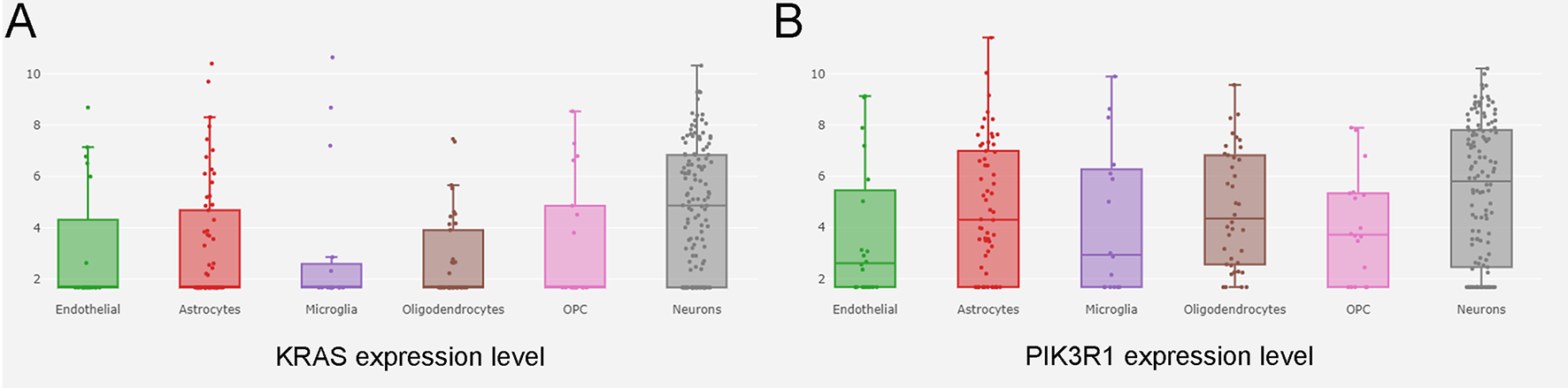

Supplement: Supplementary Figure 1 — Single-cell expression level of Kirsten rat sarcoma viral oncogene homolog (KRAS) and Phosphoinositide-3-Kinase Regulatory Subunit 1 (PIK3R1) in the healthy brain. (A,B) KRAS and PIK3R1 expression level in endothelial, astrocytes, microglia, oligodendrocytes, oligodendrocyte precursor cell (OPC), and neuron. [file Image_1.TIF]
