# Supplementary material for: Identification of Immune Hub Genes Associated With Braak Stages in Alzheimer’s Disease and Their Correlation of Immune Infiltration
Source: Front Aging Neurosci. 2022 May 10;14:887168. doi: 10.3389/fnagi.2022.887168 (PMC9129065; doi:10.3389/fnagi.2022.887168)
Supplement: Supplementary file 2 [file Table_1.DOCX]

Table S1: List of 260 Braak stages-related immune genes

| Official Gene Symbol | Official Gene Symbol | Official Gene Symbol | Official Gene Symbol | Official Gene Symbol |
| --- | --- | --- | --- | --- |
| C3 | ANXA6 | GLP2R | NMB | PTH1R |
| CD14 | AP3B1 | GMFB | NPFF | PTK2B |
| CHIT1 | APLNR | GPI | NPY | PTPN11 |
| CMTM3 | APOD | GRB2 | NR1D2 | RAC3 |
| CSF3R | AZGP1 | GREM1 | NR2F1 | RASGRP1 |
| CYBB | BCL3 | GREM2 | NR2F6 | RBP4 |
| FABP5 | BECN1 | HLA-DRB4 | NR3C1 | RELB |
| FCGR3A | BID | HLA-F | NR3C2 | RFX5 |
| FPR1 | BMP7 | HMGB1 | NR4A2 | RN7SL1 |
| HAMP | BMP8B | HSP90AA1 | NR4A3 | RORA |
| HCST | BMPR2 | HSP90AB1 | NRG3 | RORB |
| HLA-B | BPHL | HSPA1B | OAS1 | S100A16 |
| HLA-C | BRD8 | HSPA4 | OGN | S100B |
| HLA-DMA | BST2 | HSPA8 | OPRM1 | S1PR1 |
| HLA-DMB | CCK | HTR1A | PAK1 | SBDS |
| HLA-DOA | CCR10 | IFNAR1 | PAK3 | SCG2 |
| HLA-DPA1 | CD320 | IFNAR2 | PAK6 | SDC3 |
| HLA-DPB1 | CD74 | IGF1 | PCSK1 | SDC4 |
| HLA-DQA1 | CD86 | IGF2 | PCSK2 | SEMA4F |
| HLA-DRA | CHGA | IKBKG | PDGFA | SEMA5B |
| HLA-DRB3 | CHGB | IL17RD | PDGFRA | SHC3 |
| HLA-DRB5 | CLEC11A | IL4R | PDIA2 | SLIT1 |
| HLA-G | CMTM4 | IL6ST | PDIA3 | SLIT2 |
| HSPA5 | COLEC12 | INSR | PIK3CB | SOCS3 |
| IFI30 | CSPG5 | IREB2 | PIK3R1 | SOD1 |
| IFITM1 | CTSB | IRF5 | PIK3R2 | SORT1 |
| IL10RA | CX3CR1 | IRF7 | PLSCR1 | SP1 |
| IL13RA1 | CXCL1 | IRF9 | PLTP | SST |
| INPP5D | CXCL12 | ISG15 | PML | SSTR1 |
| MANF | CXCL14 | ISG20 | PMP2 | SSTR2 |
| OXTR | CXCL2 | JUN | PNOC | STAT3 |
| PTPRC | CYLD | KCNH2 | PPIA | TAP1 |
| S100A10 | DEFA3 | KITLG | PPP3CA | TAP2 |
| S100A11 | EDN1 | KLRC4 | PPP3CB | TGFB2 |
| S100A8 | EIF2AK2 | KRAS | PPP3CC | TGFB3 |
| SERPINA3 | ESRRA | LANCL1 | PPP3R1 | TGFBR2 |
| TLR2 | FABP3 | LCN15 | PRDX2 | THRB |
| TYROBP | FAM3C | LIF | PRKCB | TMSB10 |
| VIM | FGF1 | LIFR | PSMC1 | TNFRSF21 |
| ACO1 | FGF12 | LMBR1 | PSMC2 | TNFRSF25 |
| ACTG1 | FGF13 | MAP2K1 | PSMC3 | TNFRSF6B |
| ACVR1C | FGF14 | MAPK8 | PSMC4 | TNFSF10 |
| ACVRL1 | FGF7 | MAPT | PSMC5 | TPM2 |
| ADAR | FGF9 | MICA | PSMC6 | TRIM27 |
| ADCYAP1R1 | FGFRL1 | MX1 | PSMD1 | TUBB3 |
| ADIPOR1 | FLT1 | MX2 | PSMD11 | TYMP |
| ADRB1 | FOS | NAMPT | PSMD14 | UBR1 |
| ADRM1 | GBP2 | NCK2 | PSMD2 | VAV3 |
| AGER | GDF1 | NEO1 | PSMD3 | VGF |
| AKT2 | GDF10 | NFAT5 | PSMD4 | VIP |
| AKT3 | GFAP | NFKBIE | PSMD8 | VIPR1 |
| ANGPTL4 | GIPR | NFYC | PSME3 | WNT5A |
